# Seventeen Cases of Primary Hyperparathyroidism in Pregnancy: A Call for Management Guidelines

**DOI:** 10.1210/js.2018-00340

**Published:** 2019-02-20

**Authors:** Aimee Natasha DiMarco, Karim Meeran, Ioannis Christakis, Vinpreet Sodhi, Catherine Nelson-Piercy, Neil Samuel Tolley, Francesco Fausto Palazzo

**Affiliations:** 1 Department of Surgery and Cancer, Imperial College, London, United Kingdom; 2 Department of Endocrine Surgery, Hammersmith Hospital, London, United Kingdom; 3 Department of Medicine, Imperial College, London, United Kingdom; 4 Department of Endocrinology, Imperial College National Health Service Trust, London, United Kingdom; 5 Department of Endocrine and General Surgery, Kings’ College Hospital, London, United Kingdom; 6 Department of Anaesthesia, Imperial College National Health Service Trust, London, United Kingdom; 7 Department of Obstetric Medicine, Guy’s and St. Thomas’ Foundation Trust, London, United Kingdom; 8 Department of Obstetric Medicine, Imperial College National Health Service Trust, London, United Kingdom

**Keywords:** endocrine disorders in pregnancy, primary hyperparathyroidism, pregnancy

## Abstract

**Context:**

The risks of primary hyperparathyroidism (pHPT) to pregnant women and their fetuses appear to increase commensurate with serum calcium levels. The management strategy for pHPT must be adapted in pregnancy and should reflect the severity of hypercalcemia. However, no guidelines exist to assist clinicians.

**Methods:**

The experience of a high-volume multidisciplinary endocrine surgical service in treating a consecutive series of pregnant women with pHPT referred for parathyroidectomy is presented and data are compared with a nonpregnant cohort with pHPT. A review of pHPT and pregnancy outcomes in the literature is provided.

**Results:**

Seventeen pregnant women and 247 age range–matched nonpregnant women with pHPT were referred for surgery over 11 years. Mean serum calcium level was higher in the pregnant cohort (2.89 vs 2.78 mmol/L; *P* = 0.03). Preoperative localization with ultrasound succeeded in eight pregnant women (47%) and sestamibi scanning did in two of six (33% imaged preconception), compared with 84 (34%) and 102 (42%) control subjects, respectively (not significant). Parathyroidectomy was performed under general anesthesia between 12 and 28 weeks’ gestation with no adverse pregnancy outcomes resulting. Cure rate was 100% vs 96% in controls.

**Conclusion:**

pHPT in pregnancy is a threat to mother and child. Medical management may be appropriate in mild disease, but in moderate to severe disease, parathyroidectomy under general anesthesia in the second trimester is safe. Localization using ionizing radiation/MRI is unnecessary, because surgical intervention in a high-volume multidisciplinary setting has excellent outcomes. Guidelines on the topic would assist clinicians.

The routine or opportunistic laboratory estimation of serum calcium levels and well-established guidelines for the management of primary hyperparathyroidism (pHPT) provided by an international consensus panel [1] have resulted in the late stages of severe pHPT with crippling osteoporosis, fragility fractures, renal calculi, and neuropsychiatric symptoms being all but consigned to historical texts. However, the important sequelae of unrecognized and/or untreated severe pHPT in pregnancy remain. In addition to the effects of pHPT in the nonpregnant woman, one should add features exclusive to the pregnant state, such as pre-eclampsia (PET), miscarriage, intrauterine growth restriction, polyhydramnios, and neonatal hypercalcemic tetany. These sequelae raise the possibility of failure to diagnose or undertreatment of milder pHPT before conception. The complication rates of pHPT in pregnancy have been estimated at 68.7% in the mother [2] and 80% in the fetus [3] and are also likely to reflect the risk profile in moderate to severe disease.

The treatment of pHPT in pregnancy requires an awareness of the risks that attempts at treatment itself may present to the developing fetus: calcitonin has short-lasting effects, bisphosphonates cross the placenta and may harm the developing skeleton, and so are contraindicated; and the use of cinacalcet is described, but the long-term effects on the fetus or neonate are unknown. If surgery is contemplated, the selection of localization imaging must account for the risks of congenital malformation and development of malignancy posed by high doses of ionizing radiation, and there may be concern (now known to be unfounded [4]) regarding the safety of general anesthesia in pregnancy. Clinicians faced with this uncommon condition therefore are tasked with performing a risk-benefit calculation: The biochemical severity of the pHPT and harmful effects of hypercalcemia and/or hyperparathormonemia to the fetus and mother, the patient’s symptoms, gestation of the pregnancy, and previous obstetric history are set against the risks of medical and/or surgical treatment.

Multidisciplinary guidelines would assist endocrinologists, obstetricians, surgeons, and anesthetists in this difficult decision-making process and optimize treatment for both mother and child. The generation of such guidelines is complicated by the fact that most of the published literature on the topic comprises individual case reports and small series with a high chance of bias and low level of evidence. The aim of this study was to present a large series of pHPT in pregnancy, to compare the characteristics of pHPT in pregnancy with that in nonpregnant women of the same age range, and to explore the existing literature on the topic.

## 1. Methods

Data were drawn from a prospectively collected, departmental endocrine surgical database spanning 11 years (April 2007 through March 2018) at a tertiary referral teaching hospital at which >650 endocrine surgical procedures (*i.e.*, thyroid, parathyroid and adrenal) are performed annually and from a review of the published scientific literature. Patients who had undergone parathyroidectomy for pHPT in pregnancy and a control group consisting of consecutive female patients matched for age range who also were treated for pHPT with parathyroidectomy were extracted from the database. The lower limit of the age range for the control group was capped at 17 years and the upper limit at 52 years to match that of the oldest pregnant patient.

The diagnosis of pHPT in pregnancy was made on the basis of elevated serum calcium levels in the presence of an inappropriately unsuppressed PTH and, in the control group, the addition of 24-hour calcium-to-creatinine clearance ratio for the exclusion of familial hypocalciuric hypercalcemia (FHH) [5]. The alteration in renal calcium handling in pregnancy renders urinary estimation less accurate and so genetic testing for the calcium-sensing receptor mutation was used, when required, to aid cases in which there was diagnostic doubt in pregnancy.

Medical notes were examined for demographic information; past obstetric and medical history; characteristics of the pHPT; mode of presentation; symptoms and sequelae; biochemical parameters; localization studies and details of the treatment; conservative measures; pharmacological agents; details of the surgery; histopathology; and the outcomes of both the surgery and pregnancy. Notes on the pregnant patients were compared with those on the control group.

Simple descriptive statistics were used and comparisons made using the χ^2^ test. These were calculated using SPSS, version 24.0 (IBM, Armonk, NY).

A review of pHPT in pregnancy was performed using the following search terms: “hyperparathyroidism” in conjunction with “pregnancy,” “miscarriage,” and “abortion” in PubMed, Embase, Scopus, Web of Science, and ‘Google Scholar, spanning 20 years from 1998 to September 2018. Inclusion criteria were all papers reporting any case of pHPT in pregnancy and referring to their management and outcomes. Exclusion criteria were non-English language, treatment of pHPT not reported, and/or outcomes not reported. The primary outcome was miscarriage, spontaneous abortion, or stillbirth at any time in the pregnancy.

## 2. Results

### A. Case Series

#### A-1. Demographics

Between April 2007 and March 2018, 1360 parathyroidectomies were performed in the department, 17 in women referred for surgery with pHPT in pregnancy and 247 (18%) for pHPT. The patients’ median age was 44 (range, 17 to 52) years at the time of surgery. The demographics of the pregnant women are listed in Table 1; median age was 35 (range, 25 to 52) years. pHPT was diagnosed in 11 women during pregnancy; all were between 6 and 12 weeks of gestation. pHPT had been diagnosed in six before conception and they became pregnant before referral for treatment of the pHPT (patients 1, 2, 4, 7, 11, and 15).

**Table 1.** Data for the 17 Pregnant Patients With Primary Hyperparathyroidism

Patient	Age (y)	Mode of Presentation	Median Serum Corr Calcium (mmol/L)	Median Serum PTH (pmol/L)	24-Hour Urinary Calcium concentration (mmol/L)	MEN/Sporadic	Imaging		Type of Operation	Gestation at Operation	No. of Glands Removed	Total Weight of Removed Glands (g)	Outcome	Pregnancy Outcome
							Sestamibi	USS						
1	37	Incidental	3.04	15.8	ND	Sporadic	Neg	Neg	BLE	19	3.5	1.34	Cure	No complications recorded at f/u
2	35	Headache	3.0	32	ND	Sporadic	ND	Neg	MIP	18	1	4.8	Cure, recurrence at 8 y	No complications recorded at f/u
3	37	Incidental	2.63	9.8	1.96	Sporadic	ND	Neg	BLE	19	1	1.35	Cure	Emergency CS at 39 wk for PROM
4	40	Recurrent miscarriage	2.78	24.1	ND	Sporadic	Pos	Pos	MIP	MC	1	1.42	Surgery post-MC; cure	First-trimester miscarriage
5	40	Hypertension	2.74	10.1	4.6	Sporadic	ND	Neg	BLE	19	1	0.61	Cure	No complications recorded at f/u
6	41	Incidental	2.80	Not recorded	ND	Sporadic	ND	Neg	BLE	T2	1	1.2	Cure	No complications recorded at f/u
7	30	Incidental	2.56	15.9	2.26	Sporadic	Neg	Neg	BLE	24	1	0.97	Cure	No complications recorded at f/u
8	32	Incidental	3.0	Not recorded	ND	Sporadic	ND	ND	BLE	28	1	—	Cure	Uncomplicated SVD
9	36	Incidental	2.66	6.5	3.35	Sporadic	ND	Pos	BLE	19	1	2.72	Cure; bone hunger	No complications recorded at f/u
10	33	Incidental	3.3	30.9	ND	Sporadic	ND	Pos	BLE	T2	2	19.5	Cure; hypertrophic scar	No complications recorded at f/u
11	25	Known MEN1	3.16	21.7	ND	MEN1	Pos	Pos	BLE	14	3	1.78	Cure	
12	52	Incidental	3.0	25.4	ND	Sporadic	ND	Pos	BLE	21	1	1.06	Cure	Elective CS (IVF triplets)
13	37	Ureteric calculi	2.75	7	ND	Sporadic	ND	Pos	BLE	Patient declined	1	1.4	Surgery postpartum; cure	IUGR and PET
14	29	Nausea/vomiting	3.35	19.8	ND	Sporadic	ND	Pos	BLE	12	2	5.43	Cure	No complications recorded at f/u
15	32	IVF workup	2.85	6	ND	Sporadic	Neg	Neg	BLE	24	2	1.81	Cure	No complications recorded at f/u
16	34	Nausea /vomiting	2.88	13	3.45	Sporadic	ND	Pos	BLE	T2	1	0.96	Cure	Uncomplicated SVD
17	27	Nausea /vomiting			ND	Sporadic	Neg	Neg	BLE	T2	1	—	Cure	Uncomplicated SVD

Abbreviations: —, data not available; BLE, bilateral neck exploration; Corr, corrected, *i.e.* albumin-corrected; CS, cesarean delivery; f/u, follow-up; IUGR, intrauterine growth restriction; MC, miscarriage; MEN1, multiple endocrine neoplasia type 1; MIP, minimally invasive parathyroidectomy; ND, not done; Neg, negative; Pos, positive; PROM, premature rupture of membranes; SVD, spontaneous vaginal delivery; USS, ultrasound scan.

The diagnosis was prompted by symptoms or pathology in nine women. These were: recurrent miscarriage (patient 2), severe headaches (patient 4), severe gestational hypertension (patient 5), known multiple endocrine neoplasia type 1 syndrome (patient 11), ureteric calculi (patient 13), and hyperemesis (patient 14, 16 and 17), and pHPT diagnosis was determined in one patient during workup for *in vitro* fertilization (IVF) (patient 15). A comparison of the characteristics of the pregnant and control groups is provided in Table 2. The median serum calcium level was 2.89 (range, 2.56 to 3.3) mmol/L in pregnancy vs 2.78 (range, 2.35 to 3.54) mmol/L in the control group (*P* = 0.03). The median PTH level was 15.9 (range, 6.0 to 32.0) pmol/L vs 18.0 (range, 4.5 to 138) pmol/L (*P* = 0.40). Twenty-four hour urinary calcium estimation was performed in all control patients but in only five pregnant patients (median, 3.45 mmol/L; range, 1.96 to 4.6 mmol/L); results were interpreted with caution. FHH was excluded using genetic testing in one pregnant patient (patient 7).

**Table 2.** Comparators Between the Pregnant Patients and Control Subjects

	Pregnant Cohort (n = 17)	Nonpregnant Females (n = 247)	*P*
Age, median (range), y	35 (25–52)	44 (17–52)	Not calculated
No. with sporadic disease, MEN, or familial	16 sporadic, 1 MEN1	229 sporadic, 15 MEN, 3 familial	0.82
Preoperative serum corr Ca, median, mmol/L	2.89 (2.56–3.3)	2.78 (2.35–3.54)	0.03*^a^*
Preoperative serum PTH, median, pmol/L	15.9 (6–32)	18 (4.5–138)	0.40
USS localization positive, no. (%)	8 (47)	84 (34)	0.36
Sestamibi scan, positive, no. (%)	2/6 (33)	104 (42)	0.59
Type of operation, no. (%)			0.22
Bilateral exploration	13 (87)	178 (72)	
Targeted approach	2 (13)	69 (28)	
Pathology, no. (%)			0.55
Single adenoma	11 (73)	197 (80)	
Multigland disease	4 (27)	50 (20)	
Outcome	17 cured, 0 persistent disease,*^b^* 1 recurrence	237 cured, 10 persistent hypercalcemia (4%), 0 recurrences	

Abbreviations: Corr, corrected, *i.e.* albumin-corrected; MEN, multiple endocrine neoplasia; MEN1, multiple endocrine neoplasia type 1; USS, ultrasound scan.

^a^ Significant at *P* < 0.05.

^b^ Defined as failure to maintain serum calcium and PTH levels within normal limits during the 12 months postoperatively and recurrence as a normalization of biochemistry in the first 12 months, followed by elevated calcium or PTH levels thereafter.

#### A-2. pHPT management

All 17 pregnant patients were treated by a multidisciplinary team consisting of health care providers in obstetrics, obstetric medicine, endocrinology, endocrine surgery, and obstetric anesthesia. The plan, following the confirmation of diagnosis, was to control the hypercalcemia medically with hydration until the second trimester, at which point parathyroidectomy would be performed. The initial “bridging” phase of treatment comprised three components: control of hypercalcemia, fetal monitoring, and preparation for surgery. A lower tolerance of hypercalcemia and reduced threshold for admission to hospital for IV rehydration was adopted in pregnancy, with a failure to maintain serum calcium level <3 mmol/L while taking oral hydration used as a trigger. Fetal well-being was monitored with serial obstetric ultrasounds and cardiotocographic monitoring as indicated. The indication for surgery in the control group was, per international consensus guidelines [1], symptoms and/or age <50 years. Treatment approaches for the pregnant and control group are listed in Table 2.

Preparation for surgery consisted of localization with ultrasound, which was successful in locating an adenoma in eight of the pregnant patients (47%) vs 84 (34%) of the nonpregnant cohort. Sestamibi scanning was not performed in any pregnant patient; however, all six patients in whom pHPT was diagnosed before pregnancy had already undergone sestamibi scanning, which was positive in two (33%) vs 104 (42%) of controls. Neither of these differences was statistically significant (*P* = 0.36 and 0.59, respectively).

All pregnant patients were successfully bridged to surgery except one (patient 4), who miscarried before the second trimester. Patient 13 declined surgery until after delivery of her baby. Therefore, 15 patients underwent parathyroidectomy during pregnancy, 14 during the second trimester and one (patient 8) early in the third trimester due to patient preference. Surgery was performed under general anesthesia by an anesthetist with expertise in obstetrics and neck endocrine surgery. The surgical approach was a bilateral exploration in 13 cases and targeted surgery in two (in whom sestamibi and ultrasound imaging were concordant). Neuromonitoring (Medtronic, Minneapolis, MN) was not used until 2011 (none of five cases), at which point it was used selectively until 2015 (two of six cases) and subsequently in all six cases. Intraoperative PTH measurement (Future Diagnostics BV, Wijchen, the Netherlands) was used when available (12 of 17 cases) using the Miami protocol [6].

In the bilateral group, a single adenoma was removed from 11 patients, two glands in three, and 3.5 glands in one patient, with a median combined specimen weight of 1.35 g (range, 0.96 to 19.5 g); thus, the disease was judged to be multiglandular in four of 15 (27%) cases. The largest adenoma (19.38 g) was removed from patient 10 (33 years old) with pHPT diagnosed incidentally and with no family history of endocrine disease.

In the control group, the operative approach was bilateral exploration in 178 patients (72%) and targeted surgery in 69 (28%) with one gland removed in 197 (80%), two removed in 25(10%), three in 15 (6%), and 3.5 in 10 (4%); thus, multiglandular disease was found in 50 control patients (20%). Again, there was no statistical significance in this difference (*P* = 0.55). Patients aged <50 years and not already known to have a genetic syndrome resulting in pHPT were referred for genetic testing postpartum, but no new diagnoses were made.

#### A-3. Pregnancy outcomes

All patients had fetal cardiac monitoring performed by a member of the obstetric team pre- and postoperatively—once just before the induction of anesthesia and then in recovery or immediately on return to the ward. No fetal distress was recorded and no patient suffered a miscarriage as a result of receiving general anesthesia. Thereafter, 14 of the mothers who underwent surgery during pregnancy proceeded to uncomplicated deliveries of healthy babies and one (patient 3) had rupture of her membranes at 39 weeks and required an emergency cesarean delivery, resulting in a healthy baby. The patient who declined surgery (patient 13) had severe PET and delivered a baby with severe intrauterine growth restriction by emergency cesarean delivery. Parathyroidectomy was performed postpartum. Patient 4 underwent parathyroid surgery after her miscarriage.

#### A-4. pHPT outcomes

All pregnant patients were cured: the median corrected calcium level at 2 weeks postoperatively was 2.37 mmol/L and median PTH level was 2.8 pmol/L. There was no persistent disease (defined as a failure to maintain normal serum calcium and PTH levels in the 12 months postoperatively) and one recurrence (defined as pHPT after a minimum of 12 months of normal biochemistry): patient 2, who had undergone targeted surgery, presented after 5 years with biochemically mild recurrent pHPT and opted for reoperation 8 years later, at which time a second adenoma was removed. There was one case of bone hunger (patient 9) postoperatively, requiring large doses of oral calcium. This patient was discharged home after 48 hours and had no adverse consequences to her pregnancy. One patient’s scar became hypertrophic (patient 10) but was treated conservatively. Most (96%) of the control group was cured, 4% having persistent hypercalcemia and one patient had a secondary hemorrhage, requiring return to the operating room (0.4%). There was no voice change or wound infection recorded in either group. Routine postoperative laryngoscopy was normal in those patients who did not refuse it.

**Figure 1.**
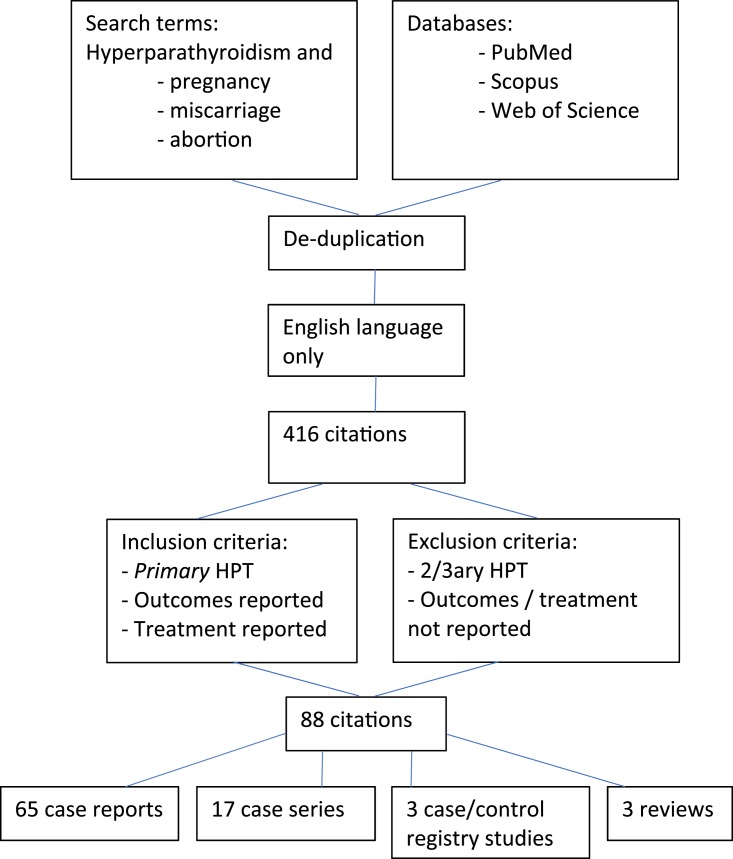
Methodology for review of literature on outcomes of pregnancy and primary hyperparathyroidism.

Figure 1 shows the search strategy used and outputs of the literature review. This produced 88 manuscripts reporting treatment and maternal and/or fetal outcomes: 3 registry-derived case-control studies, 65 case reports, 17 case series, and 3 reviews, reporting 155 individual cases and 1233 cases from registries [7].

## 3. Discussion

### A. Diagnosis of pHPT in Pregnancy

pHPT is often considered asymptomatic in pregnancy; indeed, over half of cases in this series were diagnosed incidentally. However, the nonspecific and neurocognitive symptoms of pHPT (*i.e.*, malaise, nausea, vomiting and fatigue), which may cause doctor visits and sick leave in the nonpregnant patient [8], may go uninvestigated in pregnancy, because of their overlap with normal pregnancy symptoms.

In contrast, published case reports of pHPT in pregnancy describe dramatic presentations, including maternal mortality [9], infant mortality [10], severe pancreatitis [9, 11–13], gestational hypertension [10, 11, 14–16], polyhydramnios [17] and multiple reports of neonatal hypocalcemic tetany as the presenting feature of occult maternal pHPT [18–30], suggesting that the diagnosis of pHPT is being missed or delayed before conception. The diagnosis of mild disease in pregnancy is complicated by the alterations in maternal physiology, because pregnancy results in hemodilution, hypoalbuminemia, increased transplacental calcium transport, and increased glomerular filtration, leading to relative hypercalciuria and inhibition of PTH-mediated bone resorption [31, 32]. The cumulative effect when coupled with the calcium-lowering effects of estrogen is to reduce the total serum calcium level [10, 33]. The normal range for calcium in pregnancy, therefore, probably should have a reduced upper limit to avoid missing the diagnosis of pHPT. Furthermore the urinary calcium to creatinine clearance estimation in pregnancy is considered unreliable owing to the physiological relative hypercalciuria of pregnancy that potentially may mask benign FHH [34].

### B. Prevalence of pHPT in Women of Reproductive Age

Pregnant women with pHPT accounted for 1.25% of parathyroidectomies performed in this tertiary institution over 11 years, which is somewhat greater than the published rate of <1% [10, 35–37]. The true prevalence of pHPT in pregnancy, however, is unknown because serum calcium is not measured in routine or even complicated pregnancies, nor in women who experience recurrent miscarriage [38].

An estimate of pHPT in pregnancy may be extrapolated from the prevalence of pHPT in women of reproductive age. The most reliable data from three large cohort studies [39–41] are likely to be those derived from the biochemical data held by a large health maintenance company database in Israel of ∼300,000 women aged 20 to 40 years. pHPT was diagnosed in one per 2000 women (0.05%) [41]. Probably the world’s largest parathyroidectomy service has reported that 8% of their parathyroidectomies are in women 16 and 44 years old [35], comparable to our cohort in which 10.5% are women in this age group. The greater prevalence in these surgical cohorts is most likely a reflection of an inherent referral bias rather than the true prevalence in the population at large. It is also probable that socioeconomic factors and the availability of IVF in the developed world have resulted in an increasing age at first pregnancy [42]: in the United States, the first-time pregnancy rate for women aged 35 to 39 years has increased ninefold between 1970 and 2012 [43]. It is envisaged that with the expansion of the definition of reproductive years, the incidence of pHPT in pregnant women will continue to increase. Indeed, the oldest patient with pHPT in the pregnant cohort of our series was a 52-year-old woman with triplets after IVF.

### C. Risks to the Mother and Fetus From pHPT

The general risks of end-organ damage arising from pHPT, namely, bone demineralization causing osteopenia and osteoporosis, renal calcification, and long-term increased cardiac risk, are of considerable importance to this young patient group and, per international consensus guidelines, mandate definitive surgical treatment even in the asymptomatic woman [1]. pHPT was diagnosed in six of our cohort before they conceived, but they were not referred until their biochemistry worsened during pregnancy, indicating that this guidance is not universally followed.

The plethora of case reports on pHPT in pregnancy include several of the dramatic and negative outcomes of the disease, whereas those in the large cohort studies appear to be better. The 155 cases published as individual and series [7] reported three fetal deaths [37, 44, 45], one maternal death from pancreatitis secondary to hypercalcemia 20 days postpartum, 11 other cases of maternal pancreatitis, nine hypertensive mothers, and nine with renal calculi; 29 had had previous miscarriages. There was polyhydramnios in six of the pregnancies. In nine cases, the presentation was an infant with hypocalcemic tetany.

By comparison, the Israeli data identified 124 pregnancies in 74 women affected by pHPT over 8 years (2005 to 2013) and found no difference in outcomes when comparing them with 431 women who had normocalcemic pregnancies [41]. However, the severity of hypercalcemia (mean calcium level, 2.67 mmol/L) and parathormonemia (mean PTH level, 4 pmol/L prepregnancy) was mild and significantly less than the biochemical severity in case series and reports [median calcium level, 3.1 (range, 2.02 to 4.85) mmol/L; median PTH level, 16.7 (range, 3 to 335) pmol/L] [7], and in our own series (median calcium level, 2.89 mmol/L; median PTH level, 15.9 pmol/L).

Another cohort study of 1057 women with pHPT and 3171 age-matched control subjects without, did not show an elevated rate of miscarriage in women with pHPT [40]. However, no information on the degree of biochemical severity was available and 54.5% of women actually underwent surgery for pHPT during the study period, with a significant difference in the rate of stillbirths before the diagnosis of pHPT [nine of 576 (1.6%) vs one of 481 (0.2%); OR, 7.6; 95% CI, 1.3 to 44.2; *P* = 0.023]. This may be due to a selection bias, with patients with more severe disease being selected for surgery and also at elevated risk of miscarriage due to their hypercalcemia.

PET has been estimated to occur in 25% of pHPT pregnancies [46] or sixfold more frequently than in its absence [47] and, as well as being a cause of miscarriage, stillbirth, intrauterine growth restriction, and preterm labor, is now known to have a lifelong adverse effect on maternal cardiovascular health [48–50]. The relationship between treated pHPT and PET has been reported in Sweden, with an OR of 6.89 (95% CI, 2.30 to 20.58) for the risk of PET in pHPT even if treated 2 years before pregnancy [47].

The difference in biochemical severity between the large cohort studies and the individually reported cases is likely to be at the root of the difference in outcomes reported and highlights the issues associated with the study of rare diseases such as this.

### D. Medical Management

Strategies for controlling the patient's calcium level during pregnancy must depend, therefore, on the severity of hypercalcemia. Those with mild elevation (<0.25 mmol above the upper limit of normal) may be treated with oral hydration and weekly serum calcium measurement. Inability to control the calcium should prompt admission to hospital for IV rehydration. Bisphosphonates are commonly used outside of pregnancy but are contraindicated in pregnancy because they cross the placenta, with adverse consequences reported in animal studies [51]. Reports exist showing the safety of short-term use in pregnancy [52, 53]; however, little is known of their long-term impact on the fetal skeleton and, therefore, their use should be avoided. Similarly, the calcium-sensing receptor agonist cinacalcet has been advocated to control extreme hypercalcemia in pregnancy [54–57]. However, given that the calcium-sensing receptor mutation is located not only on the chief cell of the parathyroid gland but also in multiple other tissues (*e.g.*, kidney, bone marrow and cortex, breast, thyroid C cells, the gastrointestinal tract, placenta, and some areas of the brain), it is quite possible that it may affect the fetal development of these systems, or inhibit transplacental calcium transport (given its presence there). These potential risks make its routine use difficult to approve until further data are available [58]. Calcitonin, which was administered to one patient in our study group before referral, promotes renal calcium excretion and is present in higher concentrations during pregnancy [32, 59]. It is not known to cross the placental barrier and, therefore, has been used in refractory hypercalcemia during pregnancy [60]. However, although no adverse outcomes are reported, the benefits are short lived and dubious.

### E. Localization Studies

If surgery is contemplated for pHPT in the nonpregnant woman, it is typically preceded by localization studies in the form of ultrasound for anatomical identification of possible parathyroid adenomata and sestamibi scanning for functional information. Sestamibi scanning of the parathyroids has an 80% to 99% sensitivity for a single adenoma [61] and ultrasound has 75% to 95% sensitivity [62]. 99-Tm-sestamibi is known to cross the placenta [63]; however, the total effective radiation dose of ∼5 to 6 mGy is approximately the threshold quoted by The American College of Obstetrics and Gynecology of 5 mGy, which is “unlikely to cause fetal harm” [64, 65]. Reduced-dose sestamibi “may therefore be considered” during pregnancy [65]. The low sensitivity in this case series would suggest the yield is too low to justify potential fetal harm, especially given that ultrasound revealed an adenoma in 47% of cases, and moreover, that cure is not contingent on imaging, provided surgery is undertaken by someone with expertise in four-gland exploration. CT scanning with multiple contrast phases (*e.g.*, 4DCT) results in an effective radiation dose approximately four times higher than sestamibi scanning and should therefore be avoided in pregnancy.

### F. Known Diagnosis of pHPT in the Young Woman

Women who have a diagnosis of pHPT and are planning a family should be counseled about the difficulties that pHPT during pregnancy may present. Several of the patients in our series were in this category and surgery before a planned pregnancy would have been preferable. In patients with a genetic predisposition to pHPT, such as multiple endocrine neoplasia or jaw tumor syndrome, the patient and her partner should be provided genetic and endocrinological counseling to aid family planning, given the knowledge that hypercalcemia may become harder to manage in pregnancy.

### G. Risks of Parathyroid Surgery in Pregnancy

As illustrated by the outcomes of the control group in this study (*i.e.*, cure rate of 96%, one secondary hemorrhage, no mortality, and no injury to the recurrent laryngeal nerve), parathyroidectomy in a high-volume institution should have very minimal attendant risks. Pregnant women undergoing anesthesia for nonobstetric surgery account for ∼1% to 2% of all anesthetic use, the most common indications being acute appendicitis, cholecystitis, trauma, and malignancy [4, 66]. The miscarriage rate after surgery performed under general anesthesia is reported as 5.8% overall and is highest in the first trimester, at 10.5% [66]. However, this is in the context of a background miscarriage rate of 15% in all known pregnancies, [67] and 5% in the second trimester [68]. A large study of English national hospital data, generated originally for costing purposes, reported that 47,628 pregnant women had an operation for a nonobstetric cause over a 10-year period, with 3060 operations classified as “ear, nose and throat” (including thyroid and parathyroidectomy) [4]. The number needed to harm was 356 to precipitate one stillbirth and 63 for one preterm delivery. Review of all the published case reports and registry studies over 20 years showed that pHPT was managed with surgery during pregnancy in 45% (n = 69) of cases, involving concurrent cesarean delivery in four. In none of these cases did the surgery result in an adverse fetal or maternal outcome, or preterm delivery. This is likely to reflect the differing nature of neck surgery in a relatively well patient as set against that of abdominal surgery in a woman with sepsis.

In conclusion, women diagnosed with pHPT outside of pregnancy who are planning to conceive should be advised to undergo surgery before conception, because the management of pHPT in pregnancy is more complex. During pregnancy, pHPT should be investigated in those with compatible symptoms and allied pathologies (*i.e.*, recurrent miscarriage, hypertension, PET, hyperemesis gravidarum). The risks of pHPT to the mother and fetus increase with biochemical severity: mild elevations in serum calcium (<0.25 mmol/L or <1 mg/dL above the upper limit of normal) may be managed conservatively during pregnancy with definitive surgical treatment recommended postpartum. However, in patients with moderate to severe hypercalcemia or symptoms, the risks of living with pHPT outweigh that of treatment. The benefits of surgery are the avoidance of potentially harmful medication and the definitive treatment of harmful hypercalcemia. The prevalence of multigland disease, absence of morbidity associated with four-gland exploration, and cure rate in this case series show that ionizing radiation and imaging other than ultrasound may be avoided and that surgical intervention in the form of bilateral exploration under general anesthesia in a high-volume multidisciplinary center should be the gold standard of care. pHPT in pregnancy currently lacks a multidisciplinary treatment consensus. Although it is an unusual problem, given the potential consequences to mother and fetus and the involvement of multiple medical specialties, the formation of guidelines is likely to assist clinicians in their decision-making and benefit patients.

## Abbreviations:

FHH: familial hypocalciuric hypercalcemia
IVF: *in vitro* fertilization
PET: pre-eclampsia
pHPT: primary hyperparathyroidism

## References

[1] Bilezikian JP, Brandi ML, Eastell R, Silverberg SJ, Udelsman R, Marcocci C, Potts JT Jr Guidelines for the management of asymptomatic primary hyperparathyroidism: summary statement from the Fourth International Workshop. J Clin Endocrinol Metab. 2014;99(10):3561–3569. 25162665 10.1210/jc.2014-1413 PMC5393490

[2] Schnatz PF, Thaxton S Parathyroidectomy in the third trimester of pregnancy. Obstet Gynecol Surv. 2005;60(10):672–682. 16186784 10.1097/01.ogx.0000180889.23678.fb

[3] Malekar-Raikar S, Sinnott BP Primary hyperparathyroidism in pregnancy-a rare cause of life-threatening hypercalcemia: case report and literature review. Case Rep Endocrinol. 2011;2011:520516. 22937284 10.1155/2011/520516 PMC3420708

[4] Balinskaite V, Bottle A, Sodhi V, Rivers A, Bennett PR, Brett SJ, Aylin P The risk of adverse pregnancy outcomes following nonobstetric surgery during pregnancy: estimates from a retrospective cohort study of 6.5 million pregnancies. Ann Surg. 2017;266(2):260–266. 27617856 10.1097/SLA.0000000000001976

[5] Christensen SE, Nissen PH, Vestergaard P, Heickendorff L, Brixen K, Mosekilde L Discriminative power of three indices of renal calcium excretion for the distinction between familial hypocalciuric hypercalcaemia and primary hyperparathyroidism: a follow-up study on methods. Clin Endocrinol (Oxf). 2008;69(5):713–720. 18410554 10.1111/j.1365-2265.2008.03259.x

[6] Carneiro DM, Solorzano CC, Nader MC, Ramirez M, Irvin GL III Comparison of intraoperative iPTH assay (QPTH) criteria in guiding parathyroidectomy: which criterion is the most accurate? Surgery. 2003;134(6):973–979. 14668730 10.1016/j.surg.2003.06.001

[7] DiMarco A, Meeran K, Christakis I, Sodhi V, Nelson-Piercy C, Samuel Tolley N, Palazzo FF . Data from literature review of hyperparathyroidism in pregnancy. Figshare.com data repository. Deposited on 12 December 2018 https://figshare.com/articles/Hyperparathyroidism_in_pregnancy/7447127/1.

[8] Lundgren E, Szabo E, Ljunghall S, Bergström R, Holmberg L, Rastad J Population based case-control study of sick leave in postmenopausal women before diagnosis of hyperparathyroidism. BMJ. 1998;317(7162):848–851. 9748176 10.1136/bmj.317.7162.848 PMC31094

[9] Jibhkate SN, Valand AG, Ansari S, Bharambe BM Hyperparathyroidism complicating pregnancy: a diagnostic challenge? J Postgrad Med. 2014;60(3):329–331. 25121380 10.4103/0022-3859.138825

[10] Gokkaya N, Gungor A, Bilen A, Bilen H, Gviniashvili D, Karadeniz Y Primary hyperparathyroidism in pregnancy: a case series and literature review. Gynecol Endocrinol. 2016;32(10):783–786. 27243597 10.1080/09513590.2016.1188916

[11] Dale A, Holbrook B, Sobel L, Rappaport V. Hyperparathyroidism in pregnancy leading to pancreatitis and preeclampsia with severe features. *Case Rep Obstet Gynecol.* 2017;**2017**:6061313. 10.1155/2017/6061313 PMC5405398 28487796

[12] Lee CC, Chao AS, Chang YL, Peng HH, Wang TH, Chao A Acute pancreatitis secondary to primary hyperparathyroidism in a postpartum patient: a case report and literature review. Taiwan J Obstet Gynecol. 2014;53(2):252–255. 25017280 10.1016/j.tjog.2013.01.029

[13] Kondo Y, Nagai H, Kasahara K, Kanazawa K Primary hyperparathyroidism and acute pancreatitis during pregnancy. Report of a case and a review of the English and Japanese literature. Int J Pancreatol. 1998;24(1):43–47. 9746889 10.1007/BF02787530

[14] Yilmaz BA, Altay M, Değertekin CK, Çimen AR, Iyidir ÖT, Biri A, Yüksel O, Törüner FB, Arslan M Hyperparathyroid crisis presenting with hyperemesis gravidarum. Arch Gynecol Obstet. 2014;290(4):811–814. 25027815 10.1007/s00404-014-3297-2

[15] Amaya García M, Acosta Feria M, Soto Moreno A, Dios Fuentes E, Navarro González E, Quijada Thong D, Del Valle A, Acosta Delgado D, Astorga Jiménez R Primary hyperparathyroidism in pregnancy. Gynecol Endocrinol. 2004;19(2):111–114. 15624273 10.1080/09513590400002334

[16] Haenel IV LC, Mayfield RK. Primary hyperparathyroidism in a twin pregnancy and review of fetal/maternal calcium homeostasis Am J Med Sci. 2000;319(3):191–194. 10746832 10.1097/00000441-200003000-00011

[17] Shani H, Sivan E, Cassif E, Simchen MJ Maternal hypercalcemia as a possible cause of unexplained fetal polyhydramnion: a case series. Am J Obstet Gynecol. 2008;199(4):410.e1–410.e5. 18928992 10.1016/j.ajog.2008.06.092

[18] Razavi CR, Charitou M, Marzouk M Maternal atypical parathyroid adenoma as a cause of newborn hypocalcemic tetany. Otolaryngol Head Neck Surg. 2014;151(6):1084–1085. 25325666 10.1177/0194599814555850

[19] Çakır U, Alan S, Erdeve Ö, Atasay B, Şıklar Z, Berberoğlu M, Arslan S Late neonatal hypocalcemic tetany as a manifestation of unrecognized maternal primary hyperparathyroidism. Turk J Pediatr. 2013;55(4):438–440. 24292040

[20] Korkmaz HA, Ozkan B, Terek D, Dizdarer C, Arslanoğlu S Neonatal seizure as a manifestation of unrecognized maternal hyperparathyroidism. J Clin Res Pediatr Endocrinol. 2013;5(3):206–208. 24072092 10.4274/Jcrpe.1037 PMC3814538

[21] Dinçer SI, Demir A, Kara HV, Günlüoglu MZ Thoracoscopic removal of a maternal mediastinal ectopic parathyroid adenoma causing neonatal hypocalcemia: a case report. Ann Thorac Cardiovasc Surg. 2008;14(5):325–328. 18989251

[22] Pieringer H, Hatzl-Griesenhofer M, Shebl O, Wiesinger-Eidenberger G, Maschek W, Biesenbach G Hypocalcemic tetany in the newborn as a manifestation of unrecognized maternal primary hyperparathyroidism. Wien Klin Wochenschr. 2007;119(3-4):129–131. 17347863 10.1007/s00508-006-0748-1

[23] McDonnell CM, Zacharin MR Maternal primary hyperparathyroidism: discordant outcomes in a twin pregnancy. J Paediatr Child Health. 2006;42(1-2):70–71. 16487395 10.1111/j.1440-1754.2006.00790.x

[24] Tutunculer F, Gunoz H Neonatal hypocalcemia due to asymptomatic maternal primary hyperparathyroidism. Indian Pediatr. 2005;42(3):294–295. 15817987

[25] Baretić M, Tomić Brzac H, Dobrenić M, Jakovčević A Parathyroid carcinoma in pregnancy. World J Clin Cases. 2014;2(5):151–156. 24868516 10.12998/wjcc.v2.i5.151 PMC4023310

[26] Jaafar R, Yun Boo N, Rasat R, Latiff HA. Neonatal seizures due to maternal primary hyperparathyroidism. *J Pediatr Child Health.* 2004;**40**(5-6):329. 10.1111/j.1440-1754.2004.00382.x 15151599

[27] Ip P Neonatal convulsion revealing maternal hyperparathyroidism: an unusual case of late neonatal hypoparathyroidism. Arch Gynecol Obstet. 2003;268(3):227–229. 12942255 10.1007/s00404-002-0316-5

[28] Cemeroglu AP, Böber E, Büyükgebiz A Prolonged hypocalcemia in a 2 month-old boy unmasking maternal diagnosis of primary hyperparathyroidism. J Pediatr Endocrinol Metab. 2001;14(6):785–787. 11453530 10.1515/jpem.2001.14.6.785

[29] Beattie GC, Ravi NR, Lewis M, Williams H, Blair AW, Campbell IW, Browning GG Rare presentation of maternal primary hyperparathyroidism. BMJ. 2000;321(7255):223–224. 10903659 10.1136/bmj.321.7255.223 PMC1118222

[30] Long K, Cartwright T, Sloan D, Lee C Cystic multiglandular maternal hyperparathyroidism diagnosed by neonatal hypocalcemic seizures. J Surg Case Rep. 2015;2015(3):rjv031. 25813155 10.1093/jscr/rjv031 PMC4374092

[31] Yu CKH, Sykes L, Sethi M, Teoh TG, Robinson S Vitamin D deficiency and supplementation during pregnancy. Clin Endocrinol (Oxf). 2009;70(5):685–690. 18771564 10.1111/j.1365-2265.2008.03403.x

[32] Kovacs CS Calcium and bone metabolism disorders during pregnancy and lactation. Endocrinol Metab Clin North Am. 2011;40(4):795–826. 22108281 10.1016/j.ecl.2011.08.002

[33] Kohlmeier L, Marcus R Calcium disorders of pregnancy. Endocrinol Metab Clin North Am. 1995;24(1):15–39. 7781623

[34] Ghaznavi SA, Saad NMA, Donovan LE The biochemical profile of familial hypocalciuric hypercalcemia and primary hyperparathyroidism during pregnancy and lactation: two case reports and review of the literature. Case Rep Endocrinol. 2016;2016:2725486. 27957351 10.1155/2016/2725486 PMC5120212

[35] Norman J, Politz D, Politz L Hyperparathyroidism during pregnancy and the effect of rising calcium on pregnancy loss: a call for earlier intervention. Clin Endocrinol (Oxf). 2009;71(1):104–109. 19138316 10.1111/j.1365-2265.2008.03495.x

[36] Kokrdova Z Pregnancy and primary hyperparathyroidism. J Obstet Gynaecol. 2010;30(1):57–59. 20121508 10.3109/01443610903315611

[37] McMullen TPW, Learoyd DL, Williams DC, Sywak MS, Sidhu SB, Delbridge LW Hyperparathyroidism in pregnancy: options for localization and surgical therapy. World J Surg. 2010;34(8):1811–1816. 20386905 10.1007/s00268-010-0569-2

[38] DiMarco A, Christakis I, Constantinides V, Regan L, Palazzo FF Undiagnosed primary hyperparathyroidism and recurrent miscarriage: the first prospective pilot study. World J Surg. 2018;42(3):639–645. 29349485 10.1007/s00268-017-4395-7 PMC5801386

[39] Richert L, Trombetti A, Herrmann FR, et al. Age and gender distribution of primary hyperparathyroidism and incidence of surgical treatment in a European country with a particularly high life expectancy. *Swiss Med Wkly.* 2009;**139**(27-28):400–404. 10.4414/smw.2009.12635 19629768

[40] Abood A, Vestergaard P Pregnancy outcomes in women with primary hyperparathyroidism. Eur J Endocrinol. 2014;171(1):69–76. 24743398 10.1530/EJE-13-0966

[41] Hirsch D, Kopel V, Nadler V, Levy S, Toledano Y, Tsvetov G Pregnancy outcomes in women with primary hyperparathyroidism. J Clin Endocrinol Metab. 2015;100(5):2115–2122. 25751112 10.1210/jc.2015-1110

[42] Australian Institute of Health and Welfare. Australia’s Mothers and Babies 2014–in Brief. Perinatal statistics series no. 28. Cat. no. PER 59. Canberra, ACT, Australia: Australian Institute of Health and Welfare; 2013.

[43] Saloojee H, Coovadia H Maternal age matters: for a lifetime, or longer. Lancet Glob Health. 2015;3(7):e342–e343. 25999095 10.1016/S2214-109X(15)00034-0

[44] Fouda MA Primary hyperparathyroidism and pregnancy. Saudi Med J. 2000;21(1):31–35. 11533747

[45] Kort KC, Schiller HJ, Numann PJ Hyperparathyroidism and pregnancy. Am J Surg. 1999;177(1):66–68. 10037311 10.1016/s0002-9610(98)00302-x

[46] Schnatz PF, Curry SL Primary hyperparathyroidism in pregnancy: evidence-based management. Obstet Gynecol Surv. 2002;57(6):365–376. 12140371 10.1097/00006254-200206000-00022

[47] Hultin H, Hellman P, Lundgren E, Olovsson M, Ekbom A, Rastad J, Montgomery SM Association of parathyroid adenoma and pregnancy with preeclampsia. J Clin Endocrinol Metab. 2009;94(9):3394–3399. 19531594 10.1210/jc.2009-0012

[48] Magee LA, von Dadelszen P Pre-eclampsia and increased cardiovascular risk. BMJ. 2007;335(7627):945–946. 17975257 10.1136/bmj.39337.427500.80 PMC2072036

[49] Bellamy L, Casas J-P, Hingorani AD, Williams DJ Pre-eclampsia and risk of cardiovascular disease and cancer in later life: systematic review and meta-analysis. BMJ. 2007;335(7627):974. 17975258 10.1136/bmj.39335.385301.BE PMC2072042

[50] Skjaerven R, Wilcox AJ, Klungsøyr K, Irgens LM, Vikse BE, Vatten LJ, Lie RT Cardiovascular mortality after pre-eclampsia in one child mothers: prospective, population based cohort study. BMJ. 2012;345:e7677. 23186909 10.1136/bmj.e7677 PMC3508198

[51] French AE, Kaplan N, Lishner M, Koren G Taking bisphosphonates during pregnancy. Can Fam Physician. 2003;49(OCT):1281–1282. 14594094 PMC2214129

[52] McNicholl DM, Heaney RG The safety of bisphosphonate use in pre-menopausal women on corticosteroids. Current Drug Safety. 2010;5(2):182–187. 19534636 10.2174/157488610790936178

[53] Levy S, Fayez I, Taguchi N, Han JY, Aiello J, Matsui D, Moretti M, Koren G, Ito S Pregnancy outcome following in utero exposure to bisphosphonates. Bone. 2009;44(3):428–430. 19059370 10.1016/j.bone.2008.11.001

[54] Nadarasa K, Bailey M, Chahal H, Raja O, Bhat R, Gayle C, Grossman AB, Druce MR The use of cinacalcet in pregnancy to treat a complex case of parathyroid carcinoma. Endocrinol Diabetes Metab Case Rep. 2014;2014(September):140056. 25298882 10.1530/EDM-14-0056 PMC4174590

[55] Gonzalo García I, Robles Fradejas M, Martín Macías MLA, Biain Ciganda A, Bustinza Beaskoetxea Z, Ruiz Pérez E, Fernández Matia G, Martínez Guisasola J Primary hyperparathyroidism in pregnancy treated with cinacalcet: a case report. J Obstet Gynaecol. 2018;38(1):132–134. 28760052 10.1080/01443615.2017.1325862

[56] Horjus C, Groot I, Telting D, van Setten P, van Sorge A, Kovacs CS, Hermus A, de Boer H Cinacalcet for hyperparathyroidism in pregnancy and puerperium. J Pediatr Endocrinol Metab. 2009;22(8):741–749. 19845125 10.1515/jpem.2009.22.8.741

[57] Horton WB, Stumpf MM, Coppock JD, et al. Gestational primary hyperparathyroidism due to ectopic parathyroid adenoma: case report and literature review. *J Endocr Soc*. 2017;**1**(9):1150–1155. 10.1210/js.2017-00172 PMC5686630 29264569

[58] Rubin MR, Silverberg SJ Use of cinacalcet and ^99m^Tc-sestamibi imaging during pregnancy. J Endocr Soc. 2017;1(9):1156–1159. 29264570 10.1210/js.2017-00308 PMC5689152

[59] Woodrow JP, Sharpe CJ, Fudge NJ, Hoff AO, Gagel RF, Kovacs CS Calcitonin plays a critical role in regulating skeletal mineral metabolism during lactation. Endocrinology. 2006;147(9):4010–4021. 16675524 10.1210/en.2005-1616

[60] Sato K Hypercalcemia during pregnancy, puerperium, and lactation: review and a case report of hypercalcemic crisis after delivery due to excessive production of PTH-related protein (PTHrP) without malignancy (humoral hypercalcemia of pregnancy). Endocr J. 2008;55(6):959–966. 18614854 10.1507/endocrj.k08e-092

[61] Norton KS, Johnson LW, Griffen FD, Burke J, Kennedy S, Aultman D, Li BD, Zibari G The sestamibi scan as a preoperative screening tool. Am Surg. 2002;68(9):812–815. 12356156

[62] Tublin ME, Pryma DA, Yim JH, Ogilvie JB, Mountz JM, Bencherif B, Carty SE Localization of parathyroid adenomas by sonography and technetium tc 99m sestamibi single-photon emission computed tomography before minimally invasive parathyroidectomy: are both studies really needed? J Ultrasound Med. 2009;28(2):183–190. 19168768 10.7863/jum.2009.28.2.183

[63] Gilbert WM, Newman PS, Eby-Wilkens E, Brace RA Technetium Tc 99m rapidly crosses the ovine placenta and intramembranous pathway. Am J Obstet Gynecol. 1996;175(6):1557–1562. 8987941 10.1016/s0002-9378(96)70106-0

[64] Moosvi SR, Smith S, Hathorn J, Groot-Wassink T Evaluation of the radiation dose exposure and associated cancer risks in patients having preoperative parathyroid localization. Ann R Coll Surg Engl. 2017;99(5):363–368. 10.1308/rcsann.2017.0014 PMC5449695 28462644

[65] American College of Obstetricians and Gynecologists’ Committee on Obstetric Practice Committee Opinion Number 656: Guidelines for diagnostic imaging during pregnancy. Obstet Gynecol. 2016;127(656):75–80. 10.1097/AOG.0000000000001316 26942391

[66] Nejdlova M, Johnson T Anaesthesia for non-obstetric procedures during pregnancy. Contin Educ Anaesth Crit Care Pain. 2012;12(4):203–206.

[67] Rai R, Regan L Recurrent miscarriage. Lancet. 2006;368(9535):601–611. 16905025 10.1016/S0140-6736(06)69204-0

[68] McNair T, Altman K. Miscarriage and recurrent pregnancy loss. In: *The John Hopkins Manual of Gynaecology and Obstetrics*. Philadelphia, PA: Lippincott, Williams & Wilkins; 2012:438–439.

